# Community Based Assessment of Biochemical Risk Factors for Cardiovascular Diseases in Rural and Tribal Area of Himalayan Region, India

**DOI:** 10.1155/2013/696845

**Published:** 2013-12-22

**Authors:** Ashok Kumar Bhardwaj, Dinesh Kumar, Sunil Kumar Raina, Pradeep Bansal, Satya Bhushan, Vishav Chander

**Affiliations:** ^1^Department of Community Medicine, Dr. Rajendra Prasad Government Medical College, Kangra, Himachal Pradesh 176001, India; ^2^Department of Biochemistry, Dr. Rajendra Prasad Government Medical College, Kangra, Himachal Pradesh 176001, India

## Abstract

*Context*. Evident change in nutrition and lifestyle among individuals of urban and rural areas raises suspicion for similar change in tribal area population of India. *Aim*. To study the biochemical risk factor for CVDs in rural and tribal population of Sub-Himalayan state of India. *Settings and Design*. Cross-sectional study in rural (low altitude) and tribal (high altitude) area of Himachal Pradesh, India. *Methodology*. Blood lipid profile using standard laboratory methods. *Statistical Analysis*. Chi-square test and multiple linear regression analysis. *Results*. Total of 900 individuals were studied in both areas. As per Asian criteria, obesity (BMI 27.5–30.0 kg/m^2^) was observed to be significantly high (*P* = 0.00) as 13.7% in tribal area as compared to 5.5% in rural area. Normal level of TC (<200 mg/dL) and LDL (<130 mg/dL) was observed in the majority of the population of both areas, whereas, at risk level of HDL (<40 mg/dL) was present in half of the population of both rural and tribal areas. The prevalence of borderline to high level of TGs was observed to be 60.2% and 55.2% in rural and tribal (*P* = 0.10) area, respectively. *Conclusion*. Prevalent abnormal lipid profile in tribal area demands establishment of an effective surveillance system for development of chronic diseases.

## 1. Introduction

Cardiovascular diseases (CVDs) include mainly heart attack, stroke, peripheral vascular diseases, and hypertension. Heart attack (ischemic heart diseases) alone contributes to 21.6 million disability-adjusted life years (DALYs) and ranks the third most common cause of death in the world [1]. It is well-known fact that the CVDs are the leading cause of death in urban population [2, 3]. But over the period of time, CVDs contribute significantly to mortality in rural areas of India as well [4–6]. This evident shift could be possibly due to ongoing social and economic transition. It is now a well-known fact that atherosclerosis begins in childhood and progresses in adulthood due to multiple coronary risk factors and is observed in early age of life [7]. Diet rich in salt and saturated fatty acids, physical inactivity, stress, and alcohol are known risk factors for CVDs [3]. The study of biochemical risk factors like total cholesterol (TC), high density lipoprotein (HDL), and low density lipoprotein (LDL) can assess individual predisposition towards CVDs.

Tribal population shares about 8.0% of total population of India and most of it resides Jammu and Kashmir state, Himachal Pradesh, and northeastern states (Himalayan belt) of India [8]. Like urban and rural population, socioeconomic transition is expected in tribal population as well due to the growth and development in the country. In addition, the information for biochemical risk factors of CVDs is less studied and not reported for tribal population of India. Therefore, the present study was conducted to assess the biochemical risk factors for CVDs in rural and tribal population of Himalayan belt, India.

## 2. Subjects and Methods

It was a community based cross-sectional study done at Nichar block (tribal area) of district Kinnaur and Shahpur block (rural area) of district Kangra, Himachal Pradesh, India. The studied areas were notified by the state government in rural and tribal area based on their socio-economic profile. Ninety percent of the rural population and 4.0% of tribal population shares the total 68,56,509 population of Himachal Pradesh [8]. Tribal study area had an altitude ranging from 2,320 to 6,816 meters and rural area had an altitude from 427 to 6,401 meters above the sea level. The rural and tribal area were already notified by the state government and the same operational definition was used. The study areas were selected purposely.

Assuming 30.0% prevalence of “at risk” (high level of TGs) lipid profile [9] with design effect of 1.4, the sample size of 450 individuals—more than 20 years of age—was calculated for each rural and tribal area. A community based survey was conducted in year 2010 by an independent trained field staff. From each study area, 30 clusters (village) were selected, and from each cluster the estimated individuals to be recruited were calculated by population proportion to size (PPS) method. Every cluster was hypothetically divided into four equal parts and individuals were recruited from each part for equal representation of cluster. Randomly, a house from each part was selected and all the individuals of more than 20 years of age were recruited till the sample size was met. Field staff administered a structured pretested interview based questionnaire. After the interview anthropometric assessment was done, and 5 mL overnight fasting venous blood sample was collected. Samples were centrifuged for separation of serum and transported in cryocan (liquid nitrogen) to field laboratory for biochemical analysis. Ethical clearance was sought from institute ethics committee (IEC) before the data collection. Informed consent was also sought before interview and collection of blood sample. In case of an illiterate individual thumb impression was taken by the field staff.

Studied individuals were assessed for total cholesterol (TC), high density lipoprotein (HDL), low density lipoprotein (LDL) and triglycerides (TGs). At risk level of TC, HDL, LDL, and TGs was defined according to National Cholesterol Education Program Expert Panel Adult Treatment Panel (NCEP-ATP III) guidelines [10]. Overweight and obesity were defined according to standard World Health Organization (WHO) cut-off points of body mass index (BMI) as >25 kg/m^2^ and >30 kg/m^2^, respectively. In addition, individuals were also assessed according to cut-off points recommended by the WHO for Asian populations (Obesity: ≥27.5 kg/m^2^ and Overweight: ≥23 kg/m^2^) [11]. As per standard WHO criteria, the prevalence of obesity and overweight was observed to be very low; therefore, only Asian cut-offs for BMI were reported in results section. TC, TGs, HDL, and LDL were analyzed at XL-300 fully automatic biochem analyzer of Transasia biomedical limited company. Kits of the same company were used while performing the tests [12].

Statistical analysis was done by using Epi Info 3.2.5 version [13]; chi square (*χ* ^2^) and unpaired student's *t*-test were used to compare the proportions and means, respectively. Multiple linear regression was done separately for dependent variables like TC, HDL, and LDL. Area (rural/tribal), age, sex, and BMI were considered as independent variables for regression modeling to detect significant predictors for dependent variables at 5% significance level.

## 3. Results

Total of 450 individuals from each of the rural and tribal area were studied with overall 91.5% response rate. Most of the household of rural area had joint (68.4%), and tribal area had nuclear (56.6%) type of family culture. Main town was on average 6.4 Kilometers (Km) far from the studied villages of rural area and 15.5 Km away from the villages of tribal area. Mean age was 45.5 and 48.5 years (*P* = 0.01) for males and 44.8 and 47.1 years (*P* = 0.02) for females in rural and tribal area, respectively. Mean BMI was 22.8 Kg/m^2^ in rural area and 20.9 Kg/m^2^ (*P* = 0.01) in tribal area among males, whereas for females it was 21.8 Kg/m^2^ in tribal area and 19.5 Kg/m^2^ (*P* = 0.00) in rural area. Almost 90.0% of studied individuals of both rural and tribal area were nonvegetarian. Overall, mean level of TC was 171.0 mg/L and LDL was 95.9 mg/L and they were significantly high in tribal area. Among males, as compared to rural area, the mean level of TC and LDL was observed to be significantly high as 171.5 mg/L (*P* = 0.01) and 96.8 mg/L (*P* = 0.02), respectively, in tribal area. Mean level TC was observed as 170.6 mg/L (*P* = 0.01) and LDL as 95.3 mg/L (*P* = 0.10) among females of tribal area. Overall and among males (rural: 189.5 mg/L; tribal: 185.0 mg/L) and females (rural: 187.9 mg/L; tribal: 180.0 mg/L) insignificant difference was observed for mean level of TGs (Table 1).

As per standard WHO criteria, prevalence of obesity (BMI > 30 kg/m^2^) was observed as 0.2% and 2.2% (*P* = 0.00); and prevalence of overweight as 5.7% and 13.7% (*P* = 0.00) in rural and tribal area, respectively (not shown in table). Whereas, as per Asian criteria, prevalence of obesity (BMI > 27.5 kg/m^2^) was found to be high (13.7%) in tribal area and low (5.0%) in rural area (*P* = 0.00). Overall and among females of tribal area obesity was observed to be significantly more among individuals of 35 to 60 years of age, whereas overweight prevalence was observed to be high and similar in both (rural: 27.3%; tribal 27.5%) the area. Prevalence of overweight was observed significantly (*P* = 0.00) high among males (49.8%) of rural area and females (31.9%) of tribal area (Table 2). Among males of rural areas, significantly (*P* = 0.00) a high prevalence of overweight was observed as 59.2%, 60.6%, and 38.5% in respective age group of <35, 36–50, and 51–65 year, whereas among females of tribal area, overweight prevalence was observed to be significantly high across all the age groups (Table 2).

Only 4.9% of rural and 0.7% of tribal population were observed with at risk level of TC (>200 mg/dL) (*P* = 0.00). As compared to tribal area, significantly high prevalence of at risk TC was observed among males (6.0%) and females (4.3%) of rural area. Like TC, prevalence of at risk LDL level (>130 mg/dL) was found high in rural (3.8%) as compared to tribal (0.5%) area (*P* = 0.00). Like TC and LDL, prevalence of at risk HDL (<40 mg/dL) level was observed to be significantly high (*P* = 0.00) in rural (57.0%) than in tribal (48.2%) area. As compared to tribal, 59.5% of females of rural area were exposed significantly (*P* = 0.01) to at risk level of HDL (Table 3).

Though the difference was insignificant, the prevalence of at risk TGs (>150 mg/dL) was observed to be as high as 60.2% in rural and 55.2% in tribal area (*P* = 0.10). Significantly high prevalence was observed high among individuals of more than 50 years of age. Prevalence of at risk TGs was 70.8% in males and 60.9% in females of rural area and was significantly high when compared to those in males and females of tribal area. It was further observed that in rural area, the males of all age groups and females of more than 50 years of age were exposed significantly more to at risk levels of TGs (Table 3).

BMI reflects the level of physical activity and dietary pattern of an individual. Significantly, in tribal area, odds of at risk level of HDL and TGs were 1.59 (95% CI: 1.06–2.39) and 2.95 (95% CI: 1.90–4.58) among overweight individuals, respectively, whereas odds of at risk level of TGs were about 2.21 (95% CI: 1.28–3.83) times among obese individuals in tribal area. Among individuals with at risk level of HDL individuals with vegetarian diet had OR of 0.53 (95% CI: 0.28–0.99) in rural area (Table 4). Risk quantification was carried out only for at risk HDL and TGs level only because their prevalence was considerably high as compared to that of TC and LDL.

Multiple linear regression analysis was done to assess the predictor of CVDs risk factors, and it was found that the area (rural/tribal) was a significant (*P* = 0.00) predictor for TC and LDL levels. Also, BMI for LDL and age and BMI for TGs were observed as significant (*P* = 0.00) predictors.

## 4. Discussion

CVDs have been a significant public health problem not only for urban areas but also for rural areas [5, 14]. Lifestyle related risk factors like diet, physical inactivity, stress, and alcohol predispose an individual to CVDs [15]. The effect of lifestyle related risk factors can be further assessed effectively by studying biochemical changes, mainly the lipid profile which reflects diet and physical inactivity of an individual. Exposure to at risk lipid profile (more than standard cut-offs) determines the predisposition for CVDs. The present study assessed the individuals for BMI and lipid profile, mainly TC, HDL, LDL, and TGs. Overall, and in both males and females, mean BMI was reported to be within normal limits in both areas. As per BMI criteria for Asians, prevalence of obesity was observed high in tribal area as compared to that in rural area. A study carried over five urban cities of India observed prevalence of obesity as 6.8% as per the standard WHO cutoff [16]. Like five cities study, present study reported obesity prevalence more among females as compare to males of tribal area. The present study observed that about one-fourth of the population of both rural and tribal area was exposed to overweight. Five cities study in urban area also observed the same. Though the criteria of BMI and study setting were different in the present study and the five cities study [16], it can be understood that even tribal population was observed with high prevalence of overweight and obesity as observed in urban [16] and rural area.

The information is available for blood lipid profile for urban and rural population but almost none is available for tribal population in India. A study in late 1980s showed that one-fourth of rural population and half of urban population had TC level above 190 mg/dL [17]. Another study in rural area of south India in year 2005 observed that one-third of study population was exposed to borderline to high level of TC [9]. In the present study, the prevalence of at risk TC levels (>200 mg/dL) was observed to be very low in both rural and tribal area. But the mean level of TC was observed to be high in tribal and rural area, when compared with other studies [18, 19]. Likewise, as compared to another study [20], low prevalence of at risk TC was observed among both males and females of rural and tribal area in the present study.

In contrast to the present study, evidence from North India reported mean high level of LDL and HDL in studied rural and urban population [17]. In the present study both rural and tribal population were exposed to high mean level of HDL than LDL. In the studies done in rural area, the mean levels of HDL and LDL were observed to be low as compared to present study [18, 19]. The current study showed that a very less fraction of population were exposed to at risk level of LDL, but more of population of both rural and tribal area were exposed to at risk level of HDL. This was found to be similar as that reported from studies conducted in rural areas of the country [9, 18]. Prevalence of at risk level of HDL was observed to be high among both males and females in the present study of both the studied area. A similar pattern was observed among other studies carried out in rural [19] and urban population [21]. Like HDL and LDL both rural and tribal population of the present study were observed high level of mean TGs. It was observed high among both males and females. The study conducted in the rural part of the country reported high level of mean TGs [18]. As compared to other studies of urban area [21, 22], it was observed that in the present study more population—both males and females—was exposed to high level of TGs. In rural area, significantly more males of all age groups and females of more than 50 years of age were exposed to at risk levels of TGs.

Existing evidence observed that more of the population of urban and rural area was exposed to high level of lipids. The present study observed that the significantly more population of both rural and tribal area was exposed to at risk level of HDL and TGs, but very low proportion of population observed was exposed to TC and LDL. This might be possibly due to early social and economic changes in rural and tribal belt of Himalayan region. In the present study, due to different and difficult geographic conditions, the rate of change of socioeconomic development might not be as rapid as observed in earlier studied rural and urban areas of the country. Therefore, establishing effective surveillance system for chronic disease will help to understand the complex interplay of urbanization and associated factors with chronic diseases.

The study has a limitation due to clustering of data at household level and would have been affected the results in terms of similar dietary habits. The history of tobacco and alcohol abuse by a family member would also have influenced the behavior of other family members. It had been tried to minimize by inclusion of household from the entire cluster for equal representation. Physical inactivity and substance abuse was not studied considering blood lipid profile reflects lifestyle pattern, in addition inability to study the glycemia status had been limitation of study. These were not considered because the aim was to study the prevalence of abnormal lipid profile at rural and tribal area only.

## Figures and Tables

**Table 1:** Lipid profile for rural and tribal area of a Himalayan state, Himachal Pradesh, India, 2010.

Variable	Male			Female			Total		
	Rural (150)	Tribal (184)	*P* value	Rural (300)	Tribal (266)	*P* value	Rural (450)	Tribal (450)	*P* value
Mean age	45.5	48.5	0.01	44.8	47.1	0.02	45.1	47.7	0.00
BMI (Kg/m^2^)	22.8	20.9	0.01	19.5	21.8	0.00	21.0	21.5	0.10
Nonvegetarian*	93.0	92.2	0.74	87.7	86.8	0.76	90.1	88.9	0.60
Mean TC (mg/litre)	165.1	171.5	0.01	165.8	170.6	0.01	165.6	171.0	0.00
Mean LDL (mg/litre)	90.9	96.8	0.02	92.2	95.3	0.10	91.8	95.9	0.00
Mean HDL (mg/litre)	41.8	40.8	0.36	39.9	42.1	0.09	40.5	41.6	0.23
Mean TGs (mg/litre)	189.5	185.0	0.70	187.9	180.0	0.35	109.1	96.4	0.35

*χ ^2^ test.

**Table 2:** Age and sex distribution of body mass index (BMI) in rural and tribal area of a Himalayan state, Himachal Pradesh, India, 2010.

Age group	Rural *N* (%)	Tribal *N* (%)	Obesity (BMI > 27.5 kg/m^2^)			Overweight (BMI > 23.0 kg/m^2^)		
			Rural (%)	Tribal (%)	*P* value	Rural (%)	Tribal (%)	*P* value
<35	38 (25.3)	75 (40.8)	13.0	6.1	0.42*	59.2	19.2	0.00
36–50	41 (27.3)	51 (27.8)	11.8	16.9	0.46	60.6	43.1	0.08
51–65	35 (23.3)	36 (19.5)	3.7	4.9	0.98*	38.5	16.0	0.05
>65	36 (24.1)	22 (11.9)	3.1	1.9	0.10*	20.3	5.6	0.22*
Subtotal (Male)	**150 (100)**	**184 (100)**	**9.3**	**7.4**	**0.57**	**49.8**	**21.1**	**0.00**

<35	119 (39.6)	119 (44.7)	0.4	17.2	0.00	7.1	28.0	0.00
36–50	64 (21.4)	77 (28.9)	1.5	21.3	0.00	10.6	37.2	0.00
51–65	89 (29.6)	46 (17.3)	2.8	19.0	0.00	10.2	35.7	0.00
>65	28 (9.4)	24 (9.1)	0.0	12.9	0.00	2.2	25.8	0.06*
Subtotal (Female)	**300 (100)**	**266 (100)**	**1.4**	**18.0**	**0.00**	**8.5**	**31.9**	**0.00**

<35	157 (34.8)	194 (43.1)	6.1	12.9	0.04	30.6	24.6	0.22
36–50	105 (23.3)	128 (28.4)	6.6	19.5	0.00	35.1	39.6	0.47
51–65	124 (27.5)	82 (18.2)	3.1	13.5	0.00	21.0	28.0	0.24
>65	64 (14.2)	46 (10.2)	1.8	7.8	0.91*	12.7	16.4	0.47

Total	450 (100)	450 (100)	5.0	13.7	0.00	27.3	27.5	0.94

*Yate's corrected chi-square test.

**Table 3:** Age and sex distribution of lipid profile in rural and tribal area of a Himalayan state, Himachal Pradesh, India, 2010.

Age group (years)	Rural (*N*)	Tribal (*N*)	TC > 200 mg/L			LDL > 130 mg/L			HDL < 40 mg/L			TG > 150 mg/L		
			Rural (%)	Tribal (%)	*P* value	Rural (%)	Tribal (%)	*P* value	Rural (%)	Tribal (%)	*P* value	Rural (%)	Tribal (%)	*P* value
<35	38 (25.3)	75 (40.8)	5.3	0.0	—	2.6	0.0	NA	52.0	46.2	0.64	64.9	50.7	0.00
36–50	41 (27.3)	51 (27.8)	4.9	2.0	0.84*	4.9	2.0	0.84*	56.1	52.9	0.76	76.5	58.5	0.00
51–65	35 (23.3)	36 (19.5)	2.9	0.0	—	5.7	2.8	0.98*	60.0	41.7	0.12	71.4	55.6	0.00
>65	36 (24.1)	22 (11.9)	11.4	0.0	0.27*	2.9	0.0	NA	45.7	26.3	0.19	40.0	21.1	0.00
Subtotal (male)	**150 (100)**	**184 (100)**	**6.0**	**0.5**	0.00*	**4.0**	**1.1**	0.17*	**52.0**	**47.5**	**0.39**	**70.8**	**55.8**	**0.00**

<35	119 (39.6)	119 (44.7)	1.7	0.8	1.00*	3.4	0.0	0.13*	58.0	40.3	0.00	49.6	45.4	0.18
36–50	64 (21.4)	77 (28.9)	3.1	1.3	0.87*	4.7	0.0	0.18*	62.5	58.4	0.62	58.4	57.8	0.84
51–65	89 (29.6)	46 (17.3)	6.7	0.0	0.17*	2.2	0.0	—	59.1	45.7	0.12	76.1	58.7	0.00
>65	28 (9.4)	24 (9.1)	10.7	0.0	0.29*	7.1	0.0	—	66.7	60.7	0.65	85.7	61.9	0.00
Subtotal (female)	**300 (100)**	**266 (100)**	**4.3**	**0.8**	**0.00**	**3.7**	**0.0**	**0.00**	**59.5**	**48.7**	**0.01**	**60.9**	**54.8**	**0.04**

<35	157 (34.8)	194 (43.1)	2.5	0.5	0.25*	3.2	0.0	0.04*	55.1	44.8	0.04	50.0	50.0	1.00
36–50	105 (23.3)	128 (28.4)	3.8	1.6	0.50*	4.8	0.8	0.34*	60.0	56.3	0.59	65.6	58.1	0.01
51–65	124 (27.5)	82 (18.2)	5.6	0.0	0.07*	3.2	1.2	0.65*	59.3	43.9	0.02	74.8	57.3	0.00
>65	64 (14.2)	46 (10.2)	11.1	0.0	0.05*	4.8	0.0	0.37*	52.4	47.5	0.58	60.3	42.5	0.00

Total	450 (100)	450 (100)	4.9	0.7	0.00	3.8	0.5	0.00	57.0	48.2	0.00	60.2	55.2	0.10

^∗^Yate's corrected chi-square test.

**Table 4:** Risk quantification for at risk level of HDL and TGs in rural and tribal area of a Himalayan state, Himachal Pradesh, India, 2010.

Risk factors	HDL < 40 mg/dL		TG > 150 mg/dL	
	Rural OR (95% CI)	Tribal OR (95% CI)	Rural OR (95% CI)	Tribal OR (95% CI)
Overweight (BMI > 23.0 kg/m^2^)	0.80 (0.50–1.26)	1.59 (1.06–2.39)	1.23 (0.76–1.98)	2.95 (1.90–4.58)
Obesity (BMI > 27.5 kg/m^2^)	1.52 (0.56–4.13)	1.35 (0.81–2.24)	1.72 (0.60–4.93)	2.21 (1.28–3.83)
Vegetarian	0.53 (0.28–0.99)	1.05 (0.59–1.85)	1.18 (0.66–2.11)	1.32 (0.75–2.33)

OR: odds ratio; CI: confidence interval.

## References

[1] Murray CJL, Lopez AD (1996). *Global Health Statistics*.

[2] Ramachandran A, Snehalatha C, Latha E, Vijay V, Viswanathan M (1997). Rising prevalence of NIDDM in an urban population in India. *Diabetologia*.

[3] Ramachandran A, Snehalatha C, Latha E, Satyavani K, Vijay V (1998). Clustering of cardiovascular risk factors in Urban Asian Indians. *Diabetes Care*.

[4] Jha P, Gajalakshmi V, Gupta PC (2006). Prospective study of one million deaths in India: rationale, design, and validation results. *PLoS Medicine*.

[5] Joshi R, Cardona M, Iyengar S (2006). Chronic diseases now a leading cause of death in rural India—mortality data from the Andhra Pradesh Rural Health Initiative. *International Journal of Epidemiology*.

[6] Kumar R, Kumar D, Jagnoor J, Aggarwal AK, Lakshmi PVM (2012). Epidemiological transition in a rural community of northern India: 18- year mortality surveillance using verbal autopsy. *Journal of Epidemiology & Community Health*.

[7] Gupta R, Misra A, Vikram NK (2009). Younger age of escalation of cardiovascular risk factors in Asian Indian subjects. *BMC Cardiovascular Disorders*.

[8] http://tribal.nic.in/WriteReadData/CMS/Documents/201306100104316683175RGI10june.pdf.

[9] Chow CK, Naidu S, Raju K (2008). Significant lipid, adiposity and metabolic abnormalities amongst 4535 Indians from a developing region of rural Andhra Pradesh. *Atherosclerosis*.

[10] (2002). Third Report of the National Cholesterol Education Program (NCEP) Expert Panel on Detection, Evaluation, and Treatment of High Blood Cholesterol in Adults (Adult Treatment Panel III) final report. *Circulation*.

[11] (1995). Physical status: the use and interpretation of anthropometry. Report of a WHO Expert Committee.

[13] Centre for Disease Control

[14] Kar SS, Thakur JS, Virdi NK, Jain S, Kumar R (2010). Risk factors for cardiovascular diseases: is the social gradient reversing in northern India?. *National Medical Journal of India*.

[15] Thomas A, Gaziano K, Srinath Reddy K, Paccaud F, Horton S, Chaturvedi V, Jamison DT, Breman JG, Measham AR (2006). Cardiovascular disease. *Disease Control Priorities in Developing Countries*.

[16] Singh RB, Pella D, Mechirova V (2007). Prevalence of obesity, physical inactivity and undernutrition, a triple burden of diseases during transition in a developing economy. The Five City Study Group. *Acta Cardiologica*.

[17] Chadha SL, Gopinath N, Shekhawat S (1997). Urban-rural differences in the prevalence of coronary heart disease and its risk factors in Delhi. *Bulletin of the World Health Organization*.

[18] Gupta R, Gupta HP, Kumar N, Joshi AK, Gupta VP (1994). Lipoprotein lipids and the prevalence of hyperlipidaemia in rural India. *Journal of Cardiovascular Risk*.

[19] Chow C, Cardona M, Raju PK (2007). Cardiovascular disease and risk factors among 345 adults in rural India-the Andhra Pradesh Rural Health Initiative. *International Journal of Cardiology*.

[20] Pradeepa R, Deepa R, Shanthi Rani S, Premalatha G, Saroja R, Mohan V (2003). Socioeconomic status and dyslipidaemia in a South Indian population: The Chennai Urban Population Study (CUPS 11). *National Medical Journal of India*.

[21] Gupta R, Deedwania PC, Gupta A, Rastogi S, Panwar RB, Kothari K (2004). Prevalence of metabolic syndrome in an Indian urban population. *International Journal of Cardiology*.

[22] Gupta R, Guptha S, Agrawal A, Kaul V, Gaur K, Gupta VP (2008). Secular trends in cholesterol lipoproteins and triglycerides and prevalence of dyslipidemias in an urban Indian population. *Lipids in Health and Disease*.

